# Supplementary material for: Redox-dependent rearrangements of the NiFeS cluster of carbon monoxide dehydrogenase
Source: eLife. 2018 Oct 2;7:e39451. doi: 10.7554/eLife.39451 (PMC6168284; doi:10.7554/eLife.39451)
Supplement: Supplementary file 1. [file elife-39451-supp1.docx]

**Supplementary File 1**

**Crystallographic data collection and refinement statistics**

|  | as-isolated  (batch 1) | as-isolated  (batch 2) | as-isolated  (batch 2)  Fe peak^†^ | as-isolated  (batch 2)  Ni peak^†^ |
| --- | --- | --- | --- | --- |
| Data collection |  |  |  |  |
| Space group | *P*2_1_2_1_2_1_ | *P*2_1_ | *P*2_1_ | *P*2_1_ |
| Cell dimensions |  |  |  |  |
| *a*, *b*, *c* (Å) | 65.6, 111.9, 195.3 | 64.4, 155.2, 66.6 | 64.5, 155.5, 66.6 | 64.5, 155.5, 66.6 |
| α, β, γ (°) | 90.0, 90.0, 90.0 | 90.0, 102.8, 90.0 | 90.0, 102.9, 90.0 | 90.0, 102.9, 90.0 |
| Resolution (Å)^*^ | 100 – 2.50  (2.55 – 2.50) | 100 – 1.72  (1.75 – 1.72) | 100 – 1.96  (2.00 – 1.96) | 100 – 2.10  (2.14 – 2.10) |
| *R*_sym_ (%)^*^ | 12.9 (69.7) | 7.8 (54.2) | 10.2 (65.3) | 8.3 (49.8) |
| CC_1/2_^*^ | 99.4 (67.0) | 99.7 (78.0) | 99.7 (73.8) | 99.6 (73.4) |
| < *I* / σ*I* >^*^ | 9.8 (2.0) | 12.0 (2.0) | 10.7 (2.1) | 9.4 (2.1) |
| Completeness (%)^*^ | 99.3 (99.1) | 98.7 (90.3) | 97.3 (91.6) | 97.9 (97.4) |
| Redundancy^*^ | 4.2 (4.0) | 3.9 (3.1) | 5.1 (4.5) | 3.0 (3.0) |
|  |  |  |  |  |
| Refinement |  |  |  |  |
| Resolution (Å) | 97.13 – 2.50 | 64.90 – 1.72 |  |  |
| No. reflections | 50322 | 132956 |  |  |
| *R*_work_ / *R*_free_ | 0.161/0.206 | 0.151/0.176 |  |  |
| No. atoms |  |  |  |  |
| protein | 9241 | 9610 |  |  |
| B-cluster | 16 | 16 |  |  |
| C-cluster | 18 | 34 |  |  |
| D-cluster | 4 | 4 |  |  |
| water | 275 | 1147 |  |  |
| *B*-factors |  |  |  |  |
| protein | 36.4 | 20.4 |  |  |
| B-cluster | 29.7 | 15.0 |  |  |
| C-cluster | 40.1 | 22.8 |  |  |
| D-cluster | 33.1 | 17.3 |  |  |
| water | 37.9 | 32.5 |  |  |
| R.m.s. deviations |  |  |  |  |
| Bond lengths (Å) | 0.004 | 0.006 |  |  |
| Bond angles (°) | 0.95 | 1.07 |  |  |
| Rotamer outliers | 0.31 | 0.40 |  |  |

^†^Bijvoet pairs were not merged during data processing.

^*^Values in parentheses are for the highest-resolution shell.

**Crystallographic data collection and refinement statistics (continued)**

|  | reduced  (batch 2) | reduced  (batch 2)  Fe peak^†^ | reduced  (batch 2)  Ni peak^†^ | reduced/O_2_-exposed  (batch 2) |
| --- | --- | --- | --- | --- |
| Data collection |  |  |  |  |
| Space group | *P*2_1_ | *P*2_1_ | *P*2_1_ | *P*1 |
| Cell dimensions |  |  |  |  |
| *a*, *b*, *c* (Å) | 64.6, 154.9, 66.5 | 64.7, 155.4, 66.6 | 64.7, 156.4, 66.8 | 65.2, 76.1, 76.1 |
| α, β, γ (°) | 90.0, 102.7, 90.0 | 90.0, 102.7, 90.0 | 90.0, 102.7, 90.0 | 75.8, 64.6, 64.5 |
| Resolution (Å)^*^ | 100 – 1.84  (1.88 – 1.84) | 100 – 2.06  (2.10 – 2.06) | 100 – 2.69  (2.75 – 2.69) | 100 – 2.60  (2.66 – 2.60) |
| *R*_sym_ (%)^*^ | 7.7 (77.7) | 8.1 (68.8) | 10.6 (77.7) | 10.4 (72.8) |
| CC_1/2_^*^ | 99.9 (72.8) | 99.8 (72.3) | 99.6 (69.7) | 99.6 (71.6) |
| < *I* / σ*I* >^*^ | 16.8 (2.0) | 13.4 (2.0) | 11.5 (2.1) | 10.8 (1.9) |
| Completeness (%)^*^ | 98.6 (89.7) | 96.4 (94.2) | 98.1 (98.0) | 94.0 (95.6) |
| Redundancy^*^ | 6.3 (5.0) | 4.3 (3.8) | 4.3 (4.1) | 3.7 (3.8) |
|  |  |  |  |  |
| Refinement |  |  |  |  |
| Resolution (Å) | 64.87 – 1.84 |  |  | 68.58 – 2.60 |
| No. reflections | 108650 |  |  | 34449 |
| *R*_work_ / *R*_free_ | 0.144/0.171 |  |  | 0.176/0.220 |
| No. atoms |  |  |  |  |
| protein | 9374 |  |  | 8925 |
| B-cluster | 16 |  |  | 16 |
| C-cluster | 18 |  |  | 18 |
| D-cluster | 4 |  |  | 4 |
| water | 926 |  |  | 102 |
| *B*-factors |  |  |  |  |
| protein | 26.4 |  |  | 56.5 |
| B-cluster | 20.5 |  |  | 74.4 |
| C-cluster | 27.7 |  |  | 80.7 |
| D-cluster | 23.0 |  |  | 100 |
| water | 37.6 |  |  | 40.5 |
| R.m.s. deviations |  |  |  |  |
| Bond lengths (Å) | 0.005 |  |  | 0.004 |
| Bond angles (°) | 1.09 |  |  | 0.86 |
| Rotamer outliers | 0.31 |  |  | 0.45 |

^†^Bijvoet pairs were not merged during data processing.

^*^Values in parentheses are for the highest-resolution shell.

**Crystallographic data collection and refinement statistics (continued)**

|  | C301S | C301S  Fe peak^†^ |
| --- | --- | --- |
| Data collection |  |  |
| Space group | *P*2_1_ | *P*2_1_ |
| Cell dimensions |  |  |
| *a*, *b*, *c* (Å) | 65.2, 144.2, 123.2 | 65.0, 144.0, 122.7 |
| α, β, γ (°) | 90.0, 98.7, 90.0 | 90.0, 98.7, 90.0 |
| Resolution (Å)^*^ | 100 – 2.00  (2.04 – 2.00) | 100 – 2.64  (2.70 – 2.64) |
| *R*_sym_ (%)^*^ | 15.5 (82.2) | 14.3 (56.7) |
| CC_1/2_^*^ | 99.3 (64.6) | 98.7 (73.0) |
| < *I* / σ*I* >^*^ | 6.9 (1.5) | 6.0 (1.8) |
| Completeness (%)^*^ | 97.9 (91.9) | 97.2 (97.0) |
| Redundancy^*^ | 4.4 (4.0) | 2.7 (2.6) |
|  |  |  |
| Refinement |  |  |
| Resolution (Å) | 93.27 – 2.00 |  |
| No. reflections | 148102 |  |
| *R*_work_ / *R*_free_ | 0.163/0.212 |  |
| No. atoms |  |  |
| protein | 18459 |  |
| B-cluster | 32 |  |
| C-cluster | 36 |  |
| D-cluster | 8 |  |
| water | 1546 |  |
| *B*-factors |  |  |
| protein | 25.7 |  |
| B-cluster | 20.2 |  |
| C-cluster | 25.2 |  |
| D-cluster | 22.4 |  |
| water | 34.2 |  |
| R.m.s. deviations |  |  |
| Bond lengths (Å) | 0.005 |  |
| Bond angles (°) | 0.76 |  |
| Rotamer outliers | 0.16 |  |

^†^Bijvoet pairs were not merged during data processing.

^*^Values in parentheses are for the highest-resolution shell.
