# Supplementary material for: Redox-dependent rearrangements of the NiFeS cluster of carbon monoxide dehydrogenase
Source: eLife. 2018 Oct 2;7:e39451. doi: 10.7554/eLife.39451 (PMC6168284; doi:10.7554/eLife.39451)
Supplement: Supplementary file 2. [file elife-39451-supp2.docx]

**Supplementary File 2**

**Metal content and activity of *Dv*CODH preparations**

| *Dv*CODH  Sample | Fe per  monomer  (as-isolated) | Ni per  monomer  (as-isolated) | As-isolated CO oxidation activity (µmol·min^−1^·mg^−1^) | Ni-reconstituted CO oxidation activity  (µmol·min^−1^·mg^−1^) |
| --- | --- | --- | --- | --- |
| WT batch 1 | 10.4 | 0.3 | 160 | 800 |
| WT batch 2 | 9.1 | 0.5 | 160 | 1600 |
| C301S | 13 | 0 | N/D | N/D |

Measurements were made using 4-8 technical replicates.
